# A Case of Pseudogout Following Zoledronic Acid Administration

**DOI:** 10.7759/cureus.15627

**Published:** 2021-06-13

**Authors:** Alexander C Hill, Rania Al Asmar, Adenrele A Olajide, Nesreen BenHamed

**Affiliations:** 1 Pharmacy, Marshall University, Joan C. Edwards School of Medicine, Huntington, USA; 2 Internal Medicine, Marshall University, Joan C. Edwards School of Medicine, Huntington, USA; 3 Rheumatology, Marshall University, Joan C. Edwards School of Medicine, Huntington, USA; 4 Endocrinology, Marshall University, Joan C. Edwards School of Medicine, Huntington, USA

**Keywords:** pseudogout, zoledronic acid, calcium pyrophosphate dihydrate (cppd), osteoporosis, bisphosphonates

## Abstract

Calcium pyrophosphate dihydrate (CPPD) deposition in the joints, often referred to as pseudogout, can lead to debilitating arthritis. Rare cases of pseudogout associated with bisphosphonate therapy have been reported in the literature, although the mechanism for this remains unclear. We report a case of a 53-year-old female who developed an acute pseudogout attack following administration of zoledronic acid for treatment of osteoporosis.

## Introduction

Calcium pyrophosphate dihydrate (CPPD) deposition disease, also known as pseudogout, is arthritis caused by calcium pyrophosphate crystal deposition in the joints. Risk factors for pseudogout include advanced age, previous trauma to the joint, and metabolic derangements from hyperparathyroidism [1]. Medications such as loop diuretics have been thought to cause acute attacks or flares of pseudogout as well.

Bisphosphonates, such as zoledronic acid, are well-established therapies for the treatment of osteoporosis. Common side effects of bisphosphonates include gastrointestinal issues, fatigue, arthralgia, and myalgia. As an intravenous infusion, zoledronic acid may also cause a flu-like illness with fever and muscle aches, which may last for several days but can be treated with acetaminophen.

Upon literature review, we found only eight cases of pseudogout secondary to bisphosphonate therapy, with two of those cases occurring from zoledronic acid. We report a case of pseudogout following administration of the bisphosphonate zoledronic acid.

## Case presentation

A 53-year-old female with thoracic vertebral compression fracture received an intravenous infusion of zoledronic acid 5 mg for treatment of osteoporosis. She had a past medical history significant for anxiety, depression, diabetes mellitus, gastroesophageal reflux disease (GERD), hypertension, hyperlipidemia, nonalcoholic fatty liver disease, osteoarthritis, previous pulmonary embolism, and vitamin D deficiency. Her chronic treatment regimen was alprazolam as needed, escitalopram, estradiol vaginal cream, folic acid, glycopyrrolate, ondansetron as needed for nausea, pantoprazole, ropinirole, tizanidine, and warfarin. She had documented intolerances to several medications, including multiple opioids, omeprazole, alendronate, and amoxicillin.

The immediate post-infusion period was uneventful. However, the patient experienced severe, sharp pain in her left wrist and left elbow, swelling of her wrist, and low-grade fever starting the day after the infusion. She tried ice, heat, ibuprofen, and acetaminophen for symptomatic relief. Her subjective fever resolved within two days, and while her joint pain improved somewhat over the following week, it persisted.

**Figure 1:**
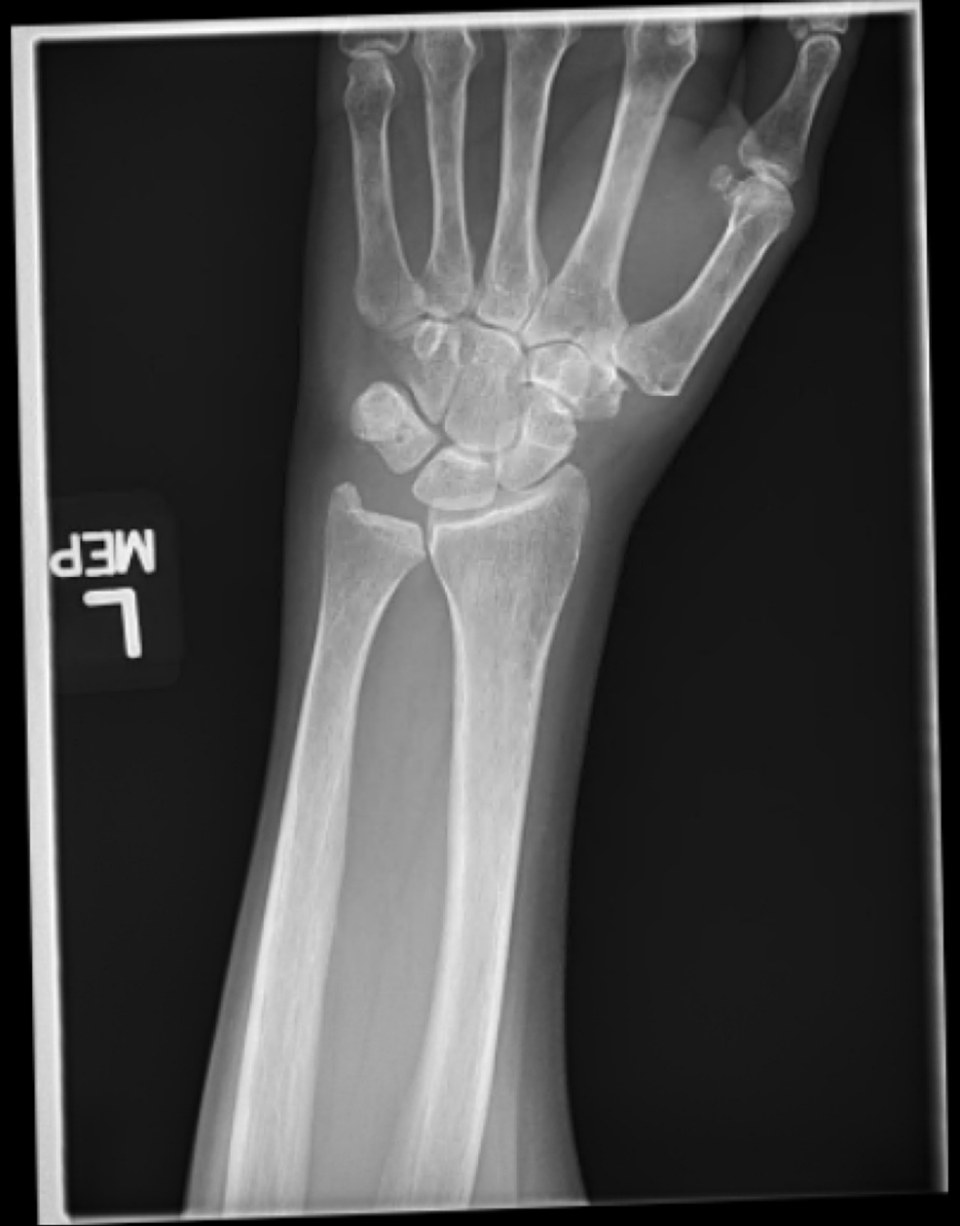
X-ray of patient's left wrist showing some basal joint arthrosis and no evidence of acute trauma.

**Figure 2:**
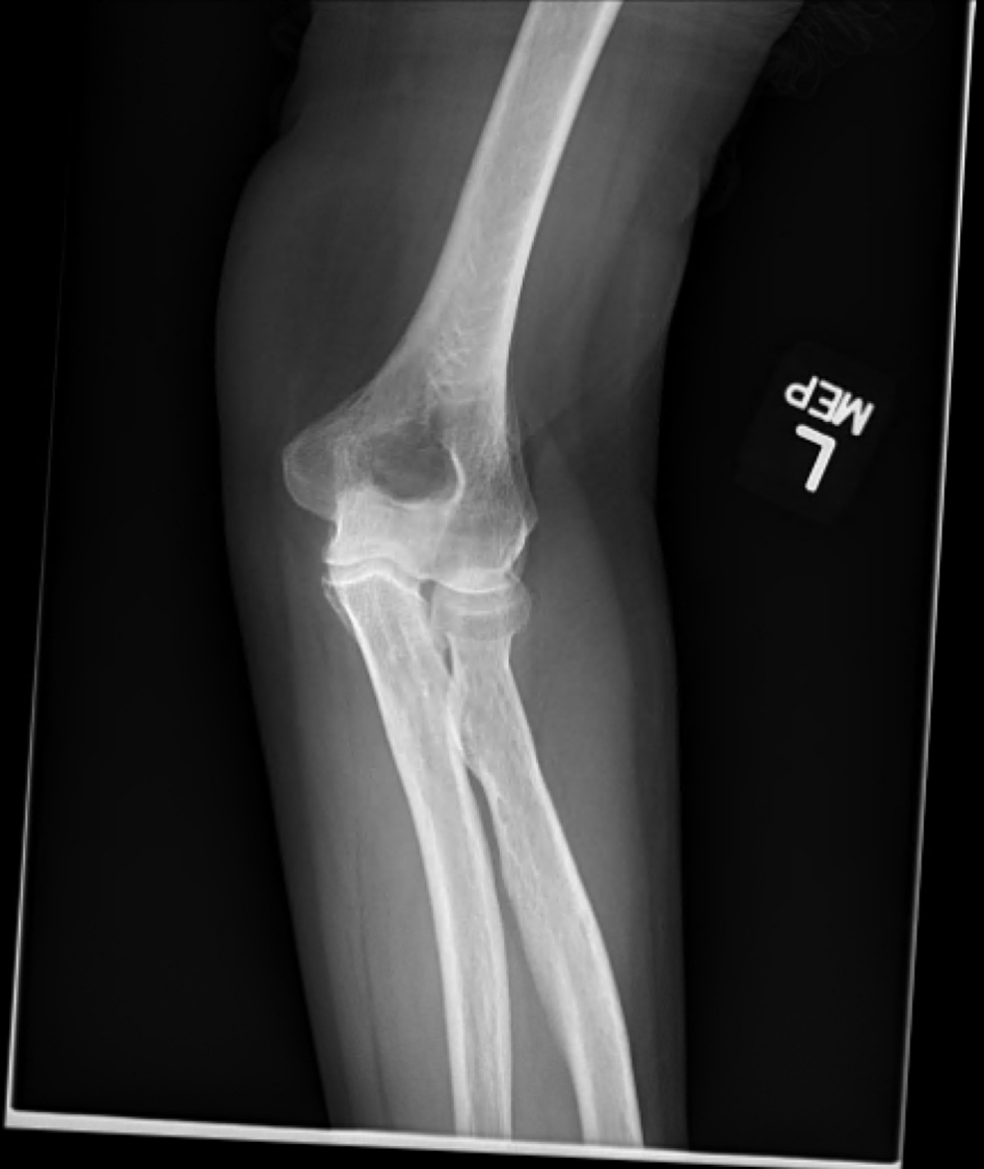
X-ray of patient's left elbow showing mild spurring around the head of the radius with no loose bodies noted.

Laboratory tests revealed elevated serum C-reactive protein (CRP) at 3.55 mg/dL (normal range 0.00 to 0.30 mg/dL) and erythrocyte sedimentation rate (ESR) at 31 mm/hr (normal range 0 to 20 mm/hr). Serum white blood cell count, calcium, and uric acid were all within normal limits. Her previous records showed that older measurements of her serum uric acid levels were also within normal limits. X-ray of the left wrist showed some basal joint arthrosis and no evidence of acute trauma (Figure 1). X-ray of the left elbow showed a spur around the head of the radius with no loose bodies noted (Figure 2). These findings did not suggest clinically significant osteoarthritis to explain her pain. The patient then saw an orthopedic specialist. At that time, she did not have any documented fever, no significant findings of joint redness or overlying skin changes, and only mild tenderness in the left wrist joint. That made the diagnosis of septic arthritis or acute gout far less likely. Thus the Orthopedician diagnosed her with a flare of pseudogout from the recent zoledronic acid use based on exclusion of other diagnoses. 

Over the next three weeks, the patient’s pain spontaneously improved, but her left wrist was still aggravated by physical activity. Her CRP improved to 0.59 mg/dL when rechecked 20 days following the initial measurement. To our knowledge, she has not experienced any further recurrences of acute pseudogout during the following three months. 

## Discussion

Our patient experienced symptoms of a pseudogout flare within 24 hours of her first zoledronic acid infusion. Her pain was primarily located in the left wrist and left elbow. Systemic inflammation was indicated by elevated serum CRP and ESR. She was seen by an orthopedic specialist, who recommended X-rays and felt that based on her presentation and timing of events/symptoms, she likely had a pseudogout flare due to zoledronic acid. Other potential causes of the patient’s symptoms were considered, such as gout and infection, but were thought to be unlikely due to lack of documented fever, serum white blood cell count, and uric acid level being within normal limits. Also, patient did not have clinically significant osteoarthritis on the joints' X-rays.

We found eight additional cases of pseudogout associated with bisphosphonates in the literature, two of which were associated with zoledronic acid [2-8]. These cases had some similarities with our case. Five of the cases were being treated for osteoporosis, and one was for osteoporosis prevention. Two of the cases involved the wrist joints, and six of the cases had an onset within one week of bisphosphonate administration. Some of these patients were also noted to have a history of and/or risk factors for pseudogout. Our patient was 53 years old, which is significantly younger than most of the other reported cases (age range 63-84 years, except one case aged 47 years).

The mechanism by which bisphosphonates can cause pseudogout attacks is unclear. One theory is that bisphosphonates inhibit alkaline phosphatase, which can dissolve calcium pyrophosphate crystals [9]. Another potential mechanism that has been suggested is the bisphosphonate‑induced lowering of serum calcium concentrations, which has been noted as a potential cause of crystal formation in the joints [10]. Despite the uncertain mechanism and the limited number of case reports present in the literature, a matched case-control study of adults with acute pseudogout between 1987 and 2012 in the UK-Clinical Practice Research Datalink found a positive association between bisphosphonate receipt and acute pseudogout [11].

Common side effects associated with bisphosphonates, especially zoledronic acid, include symptoms of a flu-like illness such as fatigue, fever, arthralgia, and myalgia. Since the adverse effects from bisphosphonates share commonality with the presentation of pseudogout, differentiating these two entities may be challenging. Although acute pseudogout due to bisphosphonates appears to be rare based on the number of cases reported in the literature, it should be considered as a possibility in patients presenting with these symptoms after bisphosphonate administration to avoid mismanagement. As in the case of our patient, this may become more readily apparent if other symptoms of flu-like illness such as fever resolve, but other symptoms such as arthralgia persist.

## Conclusions

To our knowledge, this is the third reported case of pseudogout associated with zoledronic acid. A growing number of cases of bisphosphonate-induced pseudogout are being reported in the literature. Although this appears to be a rare side effect, providers need to consider it in patients who present with persistent arthralgia following bisphosphonate administration so that it may be appropriately managed.

## References

[1] Rosenthal AK, Ryan LM (2016). Calcium pyrophosphate deposition disease. N Engl J Med.

[2] Couture G, Delzor F, Bagheri H, Micallef J, Ruyssen-Witrand A, Laroche M (2017). First cases of calcium pyrophosphate deposition disease after zoledronic acid therapy. Joint Bone Spine.

[3] Carda S, Invernizzi M, Sainaghi PP, Cisari C (2010). Acute pseudogout following intravenous neridronate for osteoporosis. J Rheumatol.

[4] Wendling D, Tisserand G, Griffond V, Saccomani C, Toussirot E (2008). Acute pseudogout after pamidronate infusion. Clin Rheumatol.

[5] Watanabe H, Yamada S, Anayama S, Sato E, Maekawa S, Sugiyama H, Nakajima I (2006). Pseudogout attack induced during etidronate disodium therapy. Mod Rheumatol.

[6] Young-Min SA, Herbert L, Dick M, Fordham J (2005). Weekly alendronate-induced acute pseudogout. Rheumatology (Oxford).

[7] Malnick SD, Ariel-Ronen S, Evron E, Sthoeger ZM (1997). Acute pseudogout as a complication of pamidronate. Ann Pharmacother.

[8] Gallacher SJ, Boyle IT, Capell HA (1991). Pseudogout associated with the use of cyclical etidronate therapy. Scott Med J.

[9] Shinozaki T, Pritzker KP (1996). Regulation of alkaline phosphatase: implications for calcium pyrophosphate dihydrate crystal dissolution and other alkaline phosphatase functions. J Rheumatol.

[10] Geelhoed GW, Kelly TR (1989). Pseudogout as a clue and complication in primary hyperparathyroidism. Surgery.

[11] Roddy E, Muller S, Paskins Z, Hider SL, Blagojevic-Bucknall M, Mallen CD (2017). Incident acute pseudogout and prior bisphosphonate use: matched case-control study in the UK-Clinical Practice Research Datalink. Medicine (Baltimore).

